# Supplementary figures and images for: Enhanced phosphorylation of PERK in primary cultured neurons as an autonomous neuronal response to prion infection
Source: PLoS One. 2020 Jun 1;15(6):e0234147. doi: 10.1371/journal.pone.0234147 (PMC7263615; doi:10.1371/journal.pone.0234147)

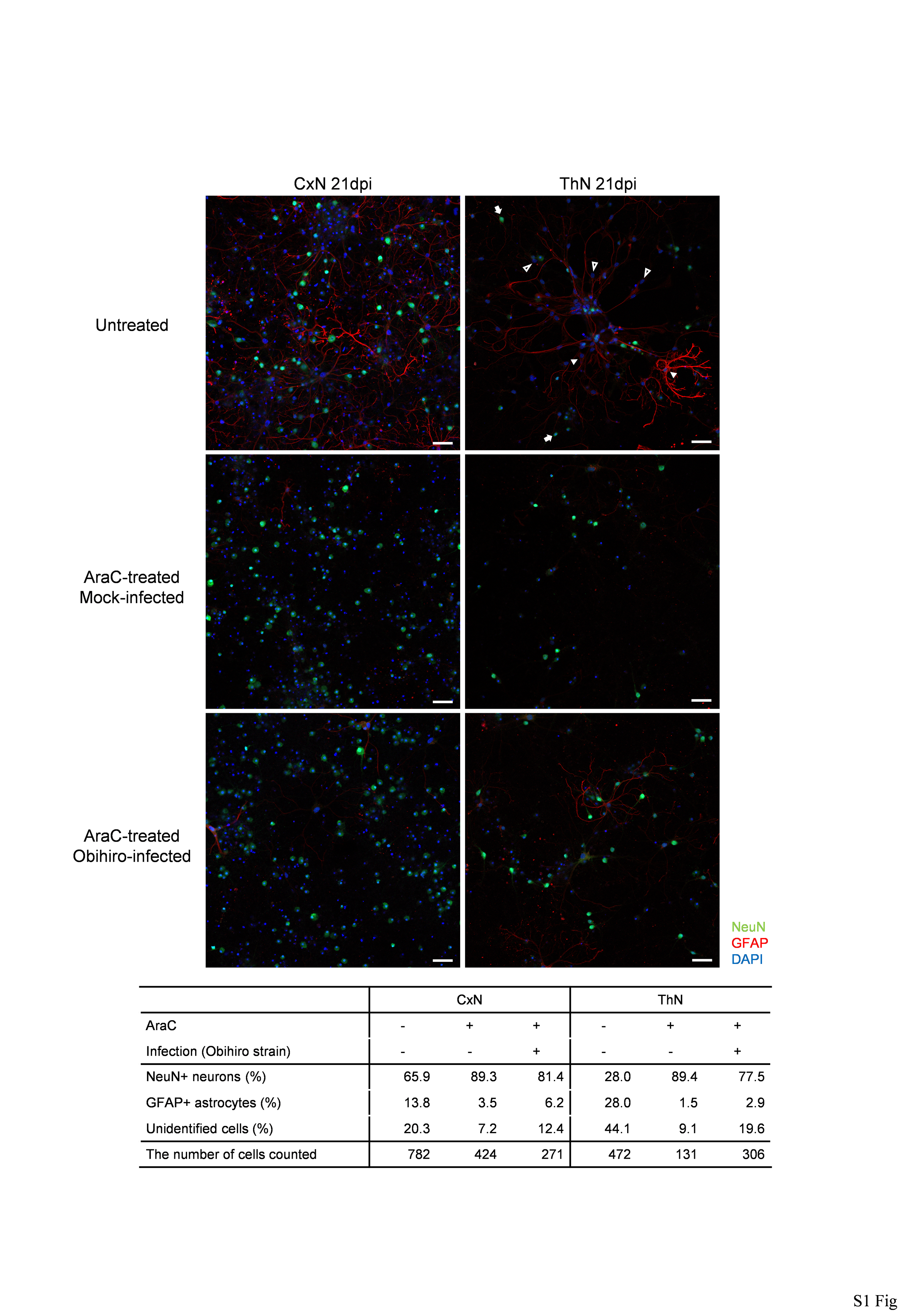

Supplement: S1 Fig — Primary neuronal cultures were treated with antimitotic AraC at 0.25 μM from 4 days in vitro (div) to 7 div in the first week. Cells were immunostained for NeuN and GFAP to identify neurons and astrocytes in the cultures at 21 dpi (corresponds to 28 div). MetaMorph software was used to set a threshold of fluorescent intensity of each cell marker. Cells which were not immunostained for NeuN and GFAP, and have shrunk and condensed nuclei (DAPI-positive area ≤ 80 μm2) were considered as putative dead cells and removed from counting. Images show representative CxNs and ThNs cultured under each condition. Arrows, filled arrowheads, and open arrowheads indicate NeuN-positive neurons, GFAP-positive astrocytes, and unidentified cells (NeuN- and GFAP-negative cells), respectively. The table shows percentages of each cell type in the cultures. Bar, 50 μm. (TIF) [file pone.0234147.s001.tif]

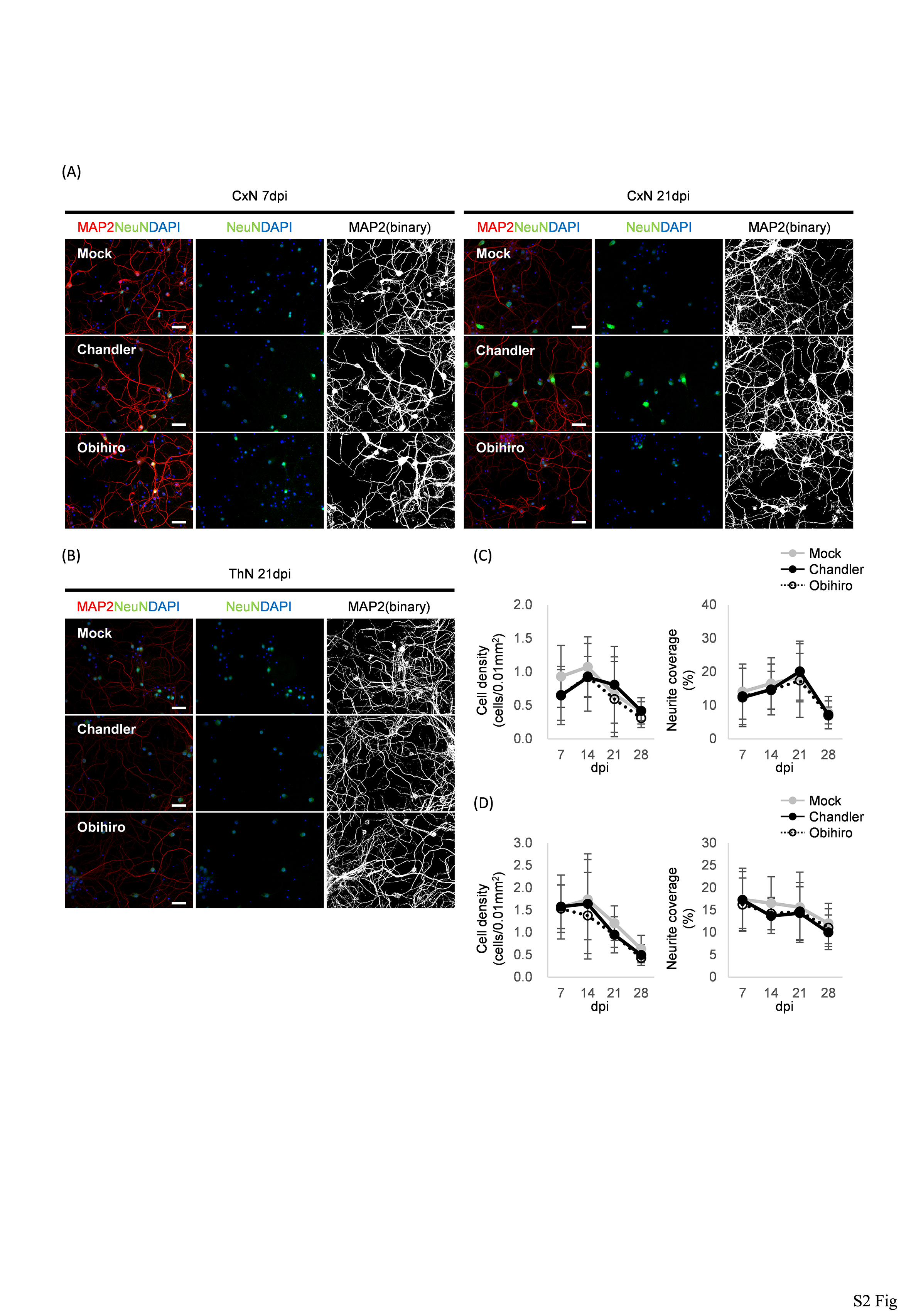

Supplement: S2 Fig — Representative immunofluorescence images of (A) CxNs and (B) ThNs used for image analyses shown in Fig 2. From the left, panels show merged images, subsets of NeuN and DAPI, and binary images of MAP2 staining. (C & D) Unnormalized values of neuronal cell density and neurite density for (C) CxNs and (D) ThNs measured by MetaMorph software. Cell density and neurite density were defined as the number of NeuN-positive nuclei per 0.01 mm2 and the surface coverage (%) by MAP2-positive neurites, respectively. Bar graphs show mean ± SD of 9 replicates from 3 independent experiments. Bars = 50 μm. (TIF) [file pone.0234147.s002.tif]

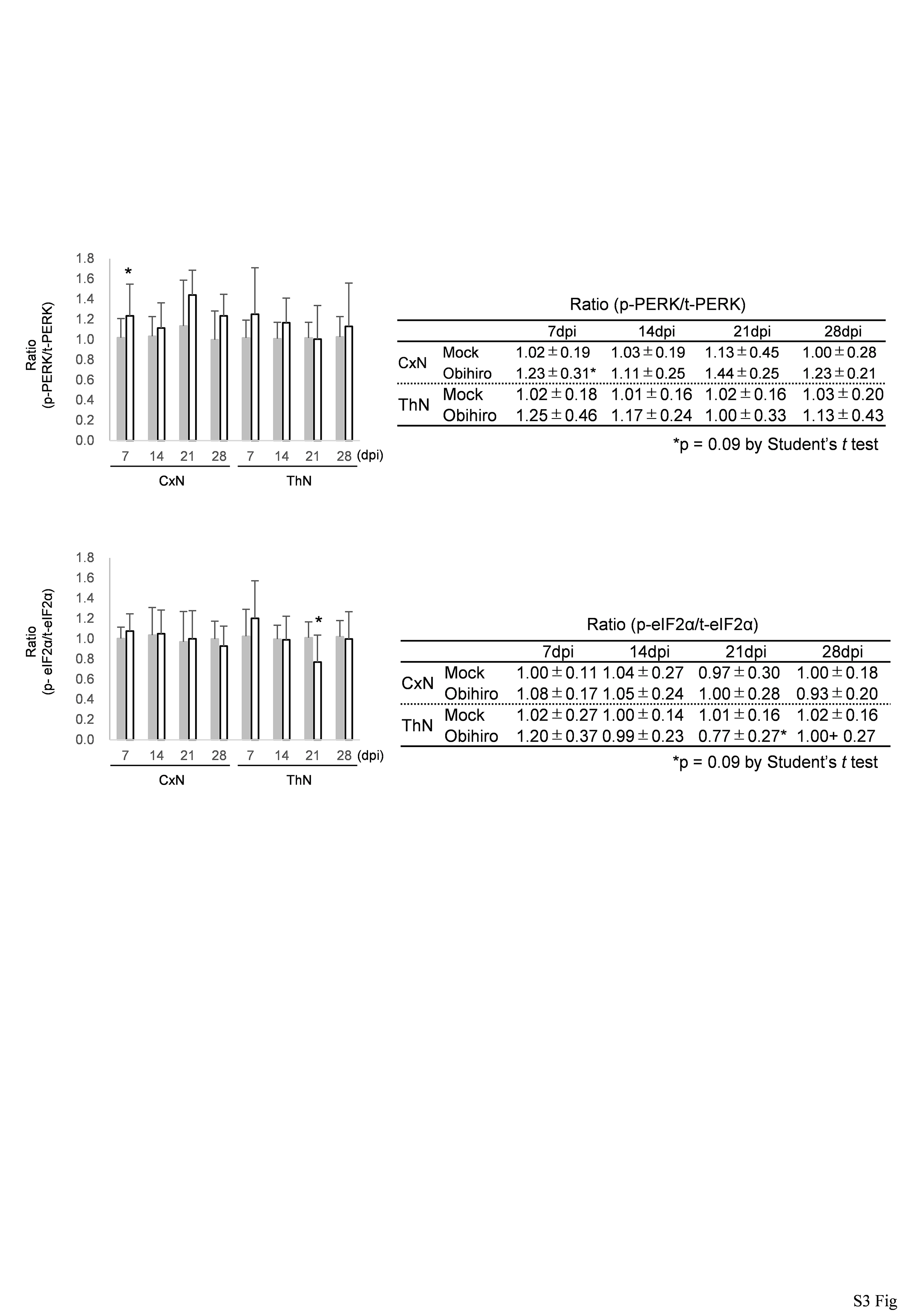

Supplement: S3 Fig — Phosphorylation levels of PERK and eIF2α were assessed as ratio between the phosphorylated and total protein signals quantified by immunoblotting described in Fig 3. Ratios of p-PERK/t-PERK (top) and p-eIF2α/ t-eIF2α (bottom) are indicated in Tables (mean ± SD). Although differences were not statistically significant, p-PERK/t-PERK ratios tended to be higher in prion-infected CxNs and ThNs than mock-infected CxNs and ThNs. In contrast to ratios of p-PERK/t-PERK, those of p-eIF2α/t-eIF2α did not differ between prion-infected and mock-infected primary neurons. (TIF) [file pone.0234147.s003.tif]

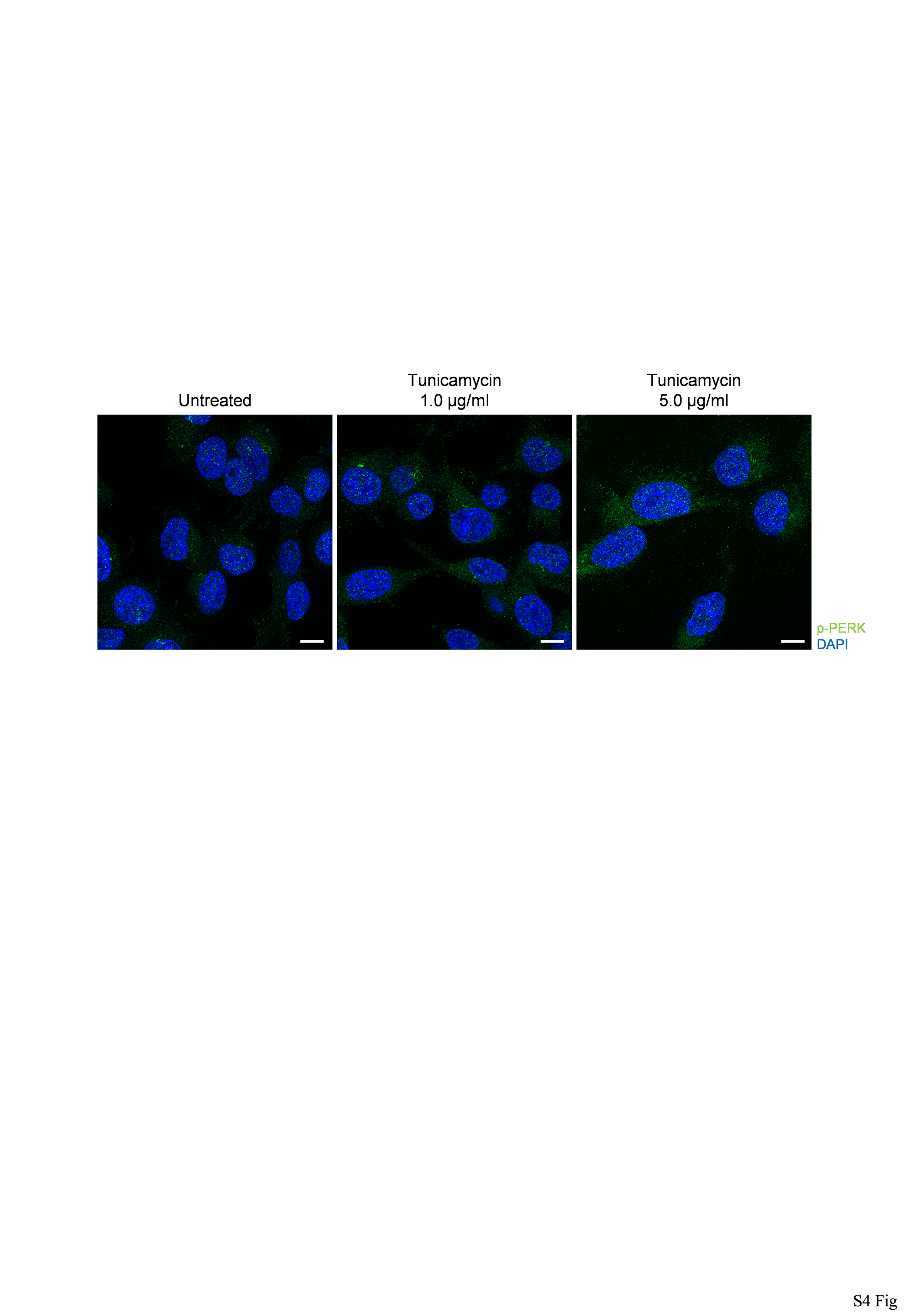

Supplement: S4 Fig — Immunofluorescence staining for p-PERK (green) with counter staining for nuclei (DAPI, blue) was carried out using Neuro2a cell treated with tunicamycin at indicated concentrations for 12 hrs. Images are shown as maximum intensity projection created from z-series stacks of confocal images. Fluorescent granular signals stained by this antibody appeared more in tunicamycin-treated Neuro2a cells (5.0 μg/ml) than in untreated Neuro2a cells, indicating that the specificity of reaction of this antibody in IFA. Bars, 10 μm. (TIF) [file pone.0234147.s004.tif]

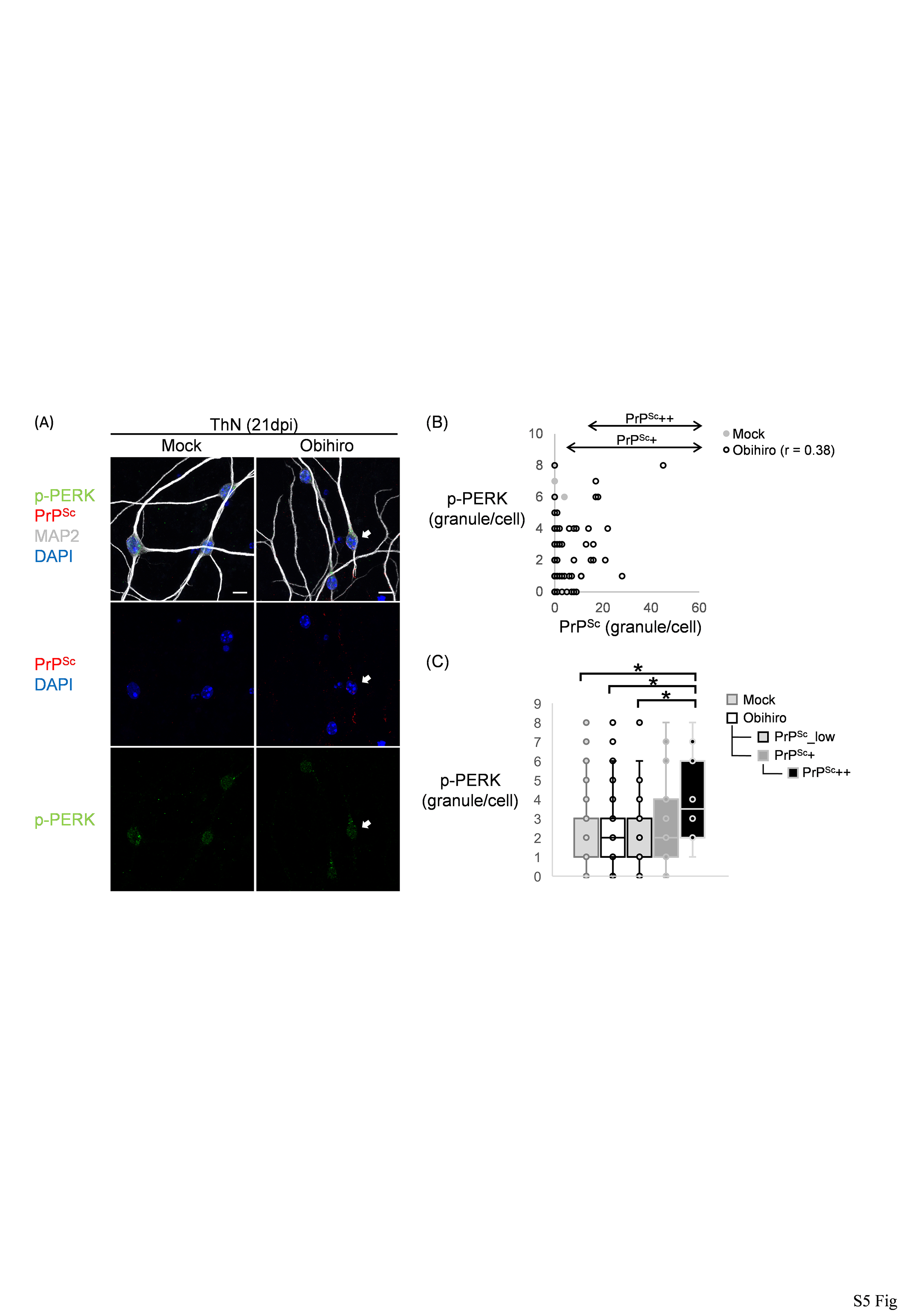

Supplement: S5 Fig — (A) Multiple immunofluorescence staining for p-PERK and PrPSc with counter staining for MAP2 and nuclei (DAPI) in ThNs at 21 dpi. PrPSc was stained with mAb 8D5. Figures are shown as maximum intensity projection images. Arrows indicate a PrPSc++ neuron. Bars, 50 μm. (B, C) Quantification of p-PERK and PrPSc in individual neurons by 3D-image analysis using Imaris software. Each dot in the scatter diagram (B) represents an individual thalamic neuron although some neurons were spotted at the same position (r, correlation coefficient). In (C), Obihiro-infected ThN was analyzed as a whole (Obihiro) and as three subpopulations classified by the frequency of PrPSc signals at the soma. The definition of the three subpopulations and the number of cells used for this analysis are as follows: PrPSc_low (< 4 PrPSc signals/cell, n = 49), PrPSc+ (≥ 4 PrPSc signals/cell, n = 26, including PrPSc++), and PrPSc++ (≥ 13 PrPSc signals/cell, n = 12). The number of cells in mock-infected ThN and Obihiro-infected ThN used for the analysis are n = 49 and n = 75, respectively. Differences between the groups were analyzed by Steel-Dwass’s multiple comparison tests. *, p < 0.05 (TIF) [file pone.0234147.s005.tif]
