# Supplementary material for: Enhanced phosphorylation of PERK in primary cultured neurons as an autonomous neuronal response to prion infection
Source: PLoS One. 2020 Jun 1;15(6):e0234147. doi: 10.1371/journal.pone.0234147 (PMC7263615; doi:10.1371/journal.pone.0234147)
Supplement: S1 Raw images — (PDF) [file pone.0234147.s006.pdf]

Original blot images used for Fig 1A

Original blot images used for Fig 1A

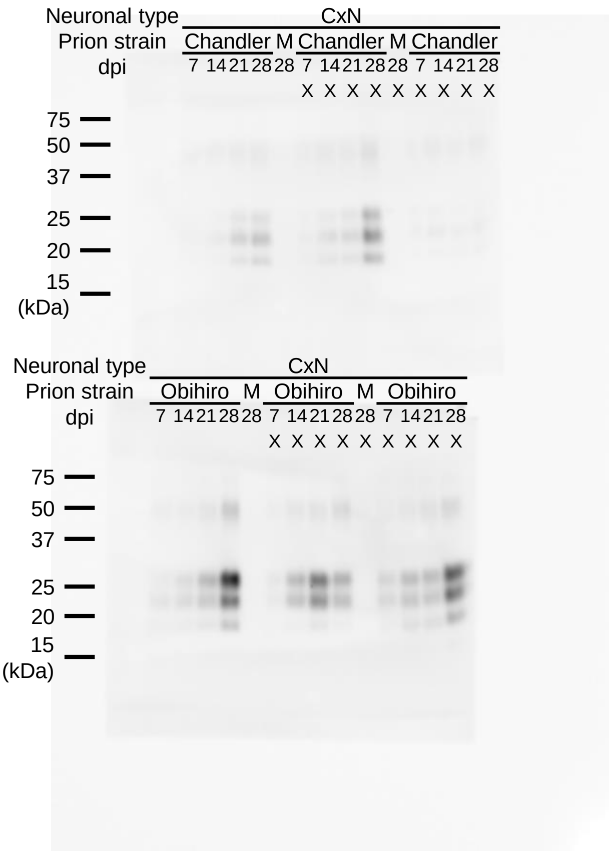

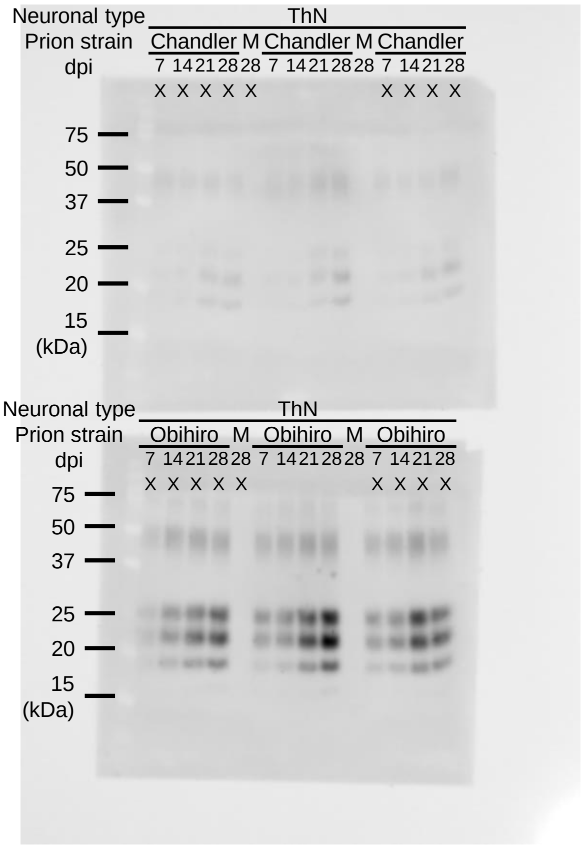

Original blot image used for Fig 1C

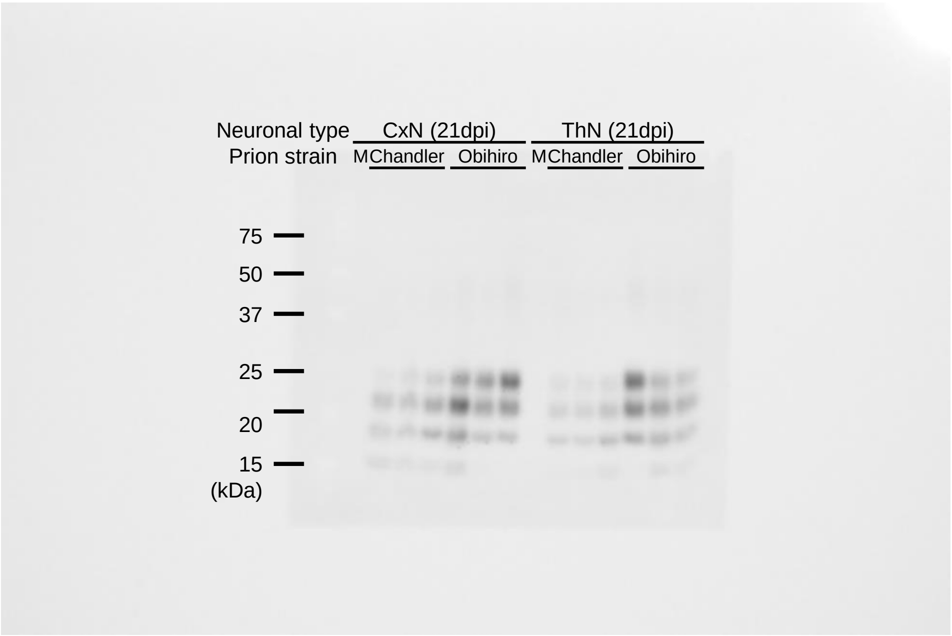

S6_raw images.

Original blot images shown in Figure 1A, 1B, and 1C. CxN, cerebral cortical neuronal culture; ThN, thalamic neuronal culture; Chandler and Obihiro, mouse-adapted scrapie strains; M, Mock-infected neurons; dpi, days post infection; X, lanes not shown in the Figure. Chemiluminescence signals were acquired by LAS-3000 chemiluminescence imager (Fuji film).

(A)

Blot 1 (14dpi, target: PERK, βIII-tubulin, eIF2α)

MWM — CxN: M O O M M O — ThN: M O M O M O — MWM

Strip 1: PERK (100-250 kDa)
Strip 2: βIII-tubulin (50 kDa)
Strip 3: eIF2α (37 kDa)

Blot 4 (21dpi, target: p-PERK, βIII-tubulin, p-eIF2α)

MWM — CxN: M M M O O O — ThN: M O M O M O — MWM

Strip 1: p-PERK (100-250 kDa)
Strip 2: βIII-tubulin (50 kDa)
Strip 3: p-eIF2α (37 kDa)

Blot 2 (14dpi, target: p-PERK, βIII-tubulin, p-eIF2α)

MWM — CxN: M O M O M O — ThN: M O M O M O — MWM

Strip 1: p-PERK (100-250 kDa)
Strip 2: βIII-tubulin (50 kDa)
Strip 3: p-eIF2α (37 kDa)

Blot 5 (28dpi, targeting PERK, βIII-tubulin, eIF2α)

MWM — CxN: M O M O M O — ThN: M O M O M O — MWM

Strip 1: PERK (100-250 kDa)
Strip 2: βIII-tubulin (50 kDa)
Strip 3: eIF2α (37 kDa)

Blot 3 (21dpi, target: PERK, βIII-tubulin, eIF2α)

MWM — CxN: M M M O O O — ThN: M O M O M O — MWM

Strip 1: PERK (100-250 kDa)
Strip 2: βIII-tubulin (50 kDa)
Strip 3: eIF2α (37 kDa)

Blot 6 (28dpi, target: p-PERK, βIII-tubulin, p-eIF2α)

MWM — CxN: M O M O M O — ThN: M O M O M O — MWM

Strip 1: p-PERK (100-250 kDa)
Strip 2: βIII-tubulin (50 kDa)
Strip 3: p-eIF2α (37 kDa)

(B)

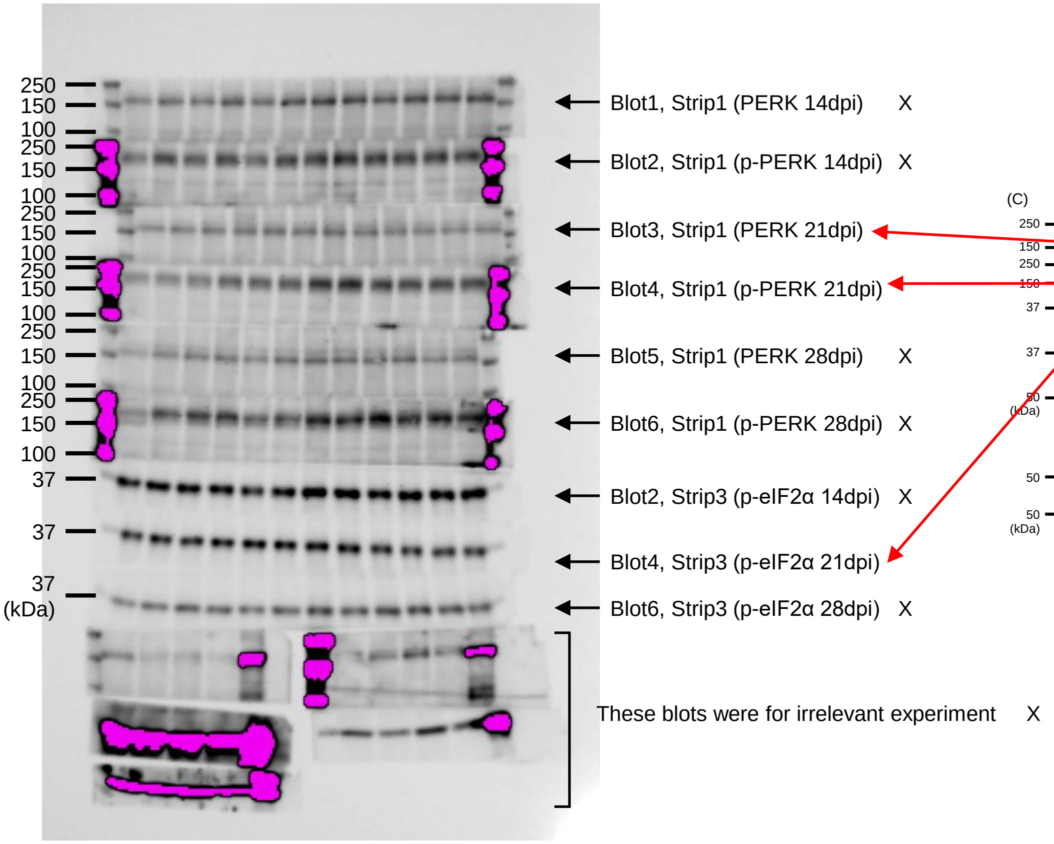

(C)

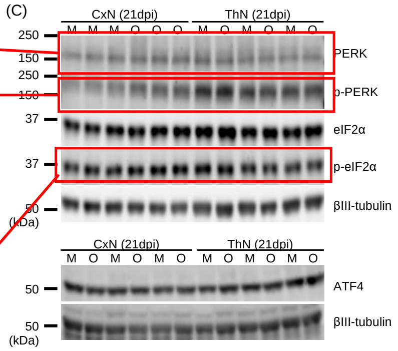

Fig. 3C

S6_raw images. (continued)

Original blot images for Fig 3C. The layouts (A) and images of original blots shown in Fig. 3C. MWM, pre-stained molecular weight marker. (B) Due to the difference of molecular weights of PERK /p-PERK, eIF2α/p-eIF2α, and βIII-tubulin, membranes were cut into three pieces according to bands of the pre-stained molecular weight marker loaded on the left- and right-most wells (at around 100 kDa and 45 kDa). Strips from a higher molecular weight range were used for the detection of PERK/p-PERK, those from a middle molecular weight range were used for the detection of βIII-tubulin, and the remaining strips with a lower molecular weight range were used for the detection of eIF2α/p-eIF2α. X, images that were not used for the blot images of Fig. 3C.

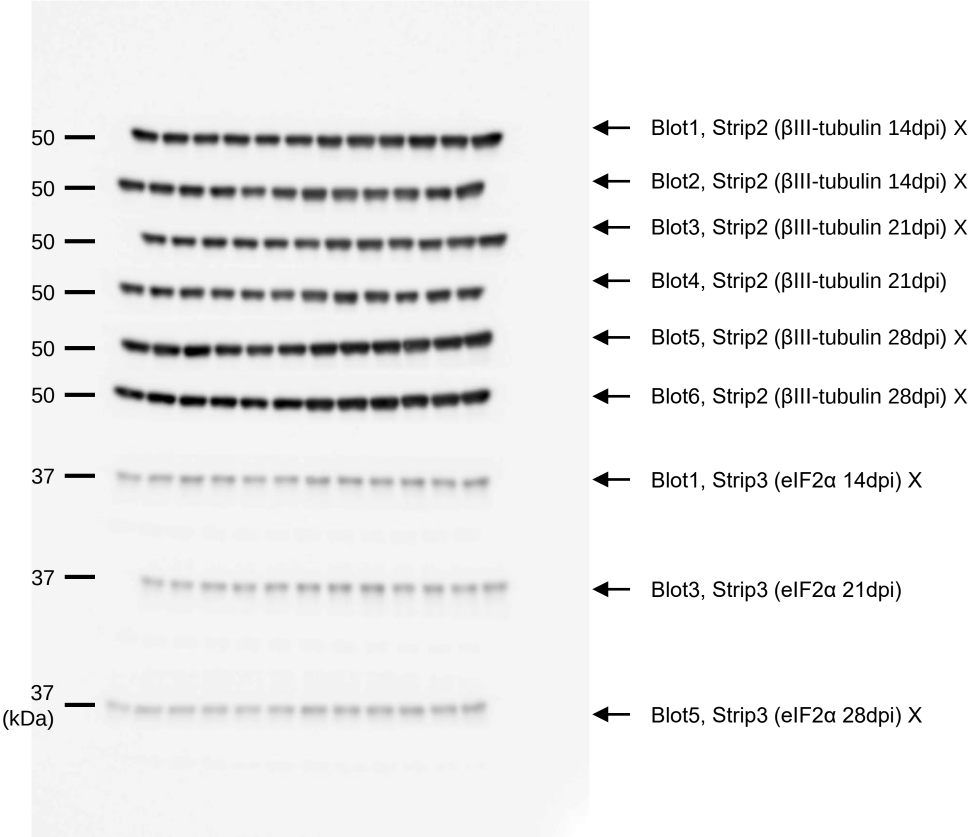

Original blot for the βIII-tubulin detection as an internal marker for eIF2α PERK detection in Fig. 3C.

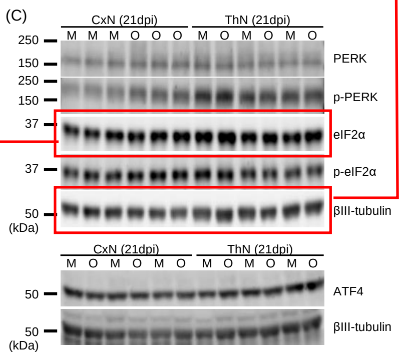

Fig. 3C

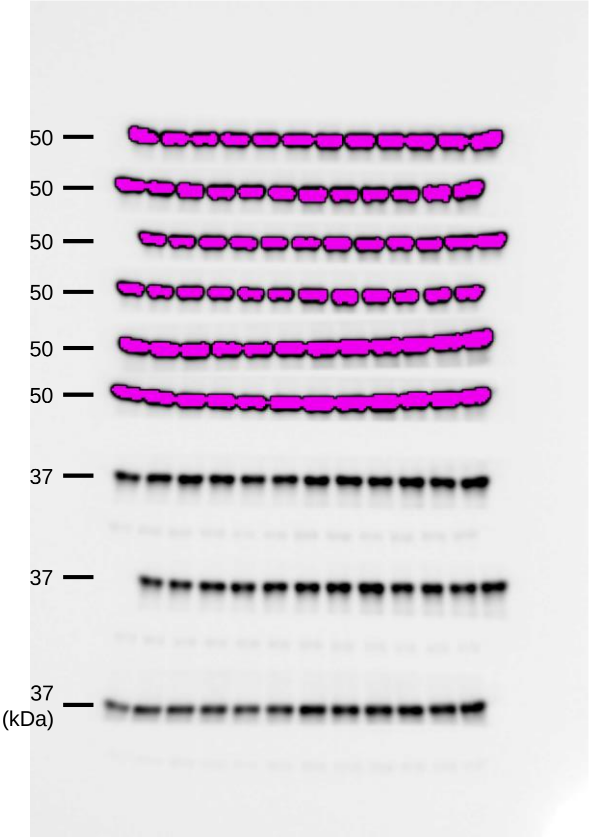

Original blot for the eIF2α detection in Fig. 3C.

Original blot images for Fig. 3C, continued.
Blot on the top were further exposed to obtain signals of eIF2α (bottom)

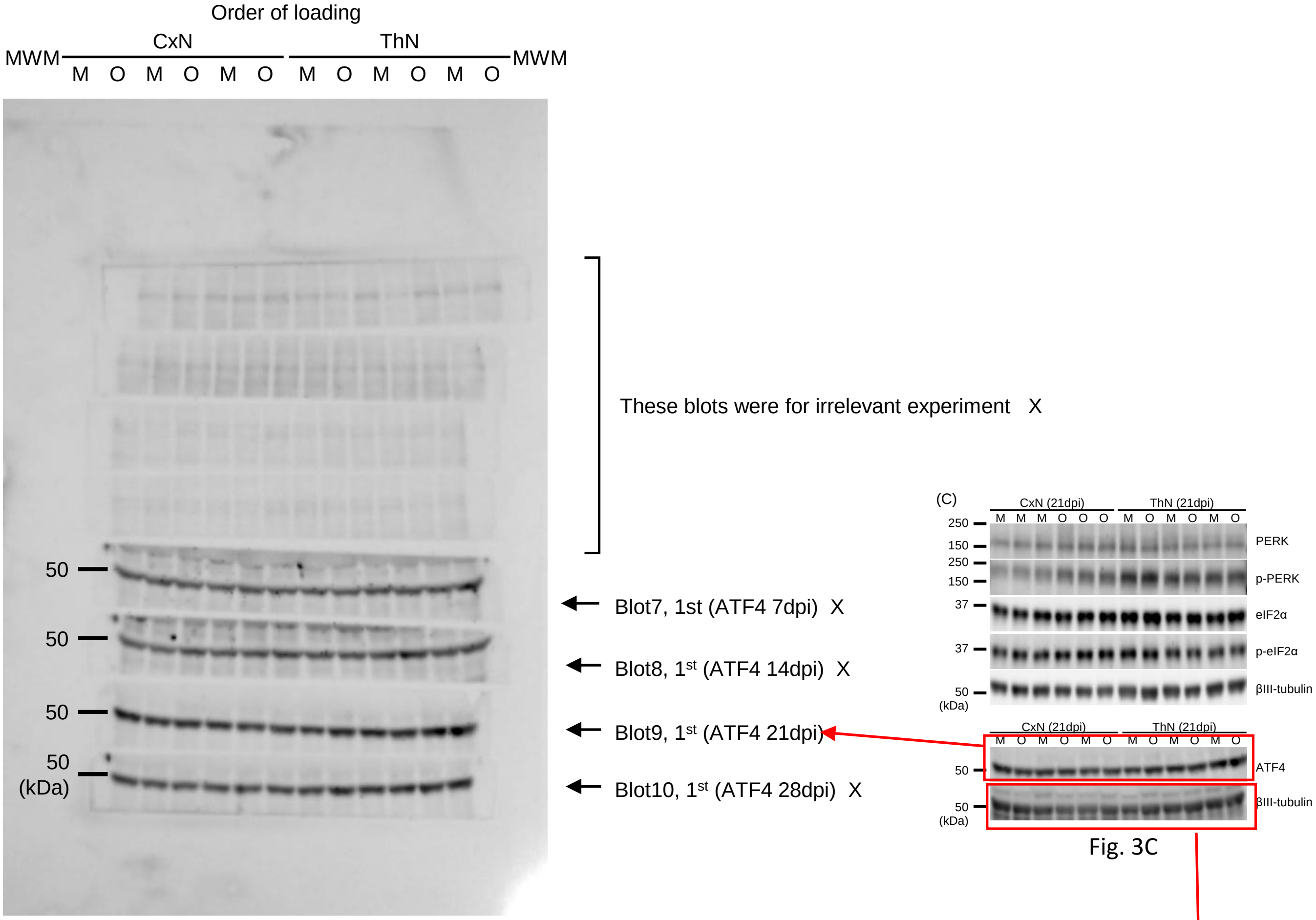

Original blot for the ATF4 detection in Fig. 3C.

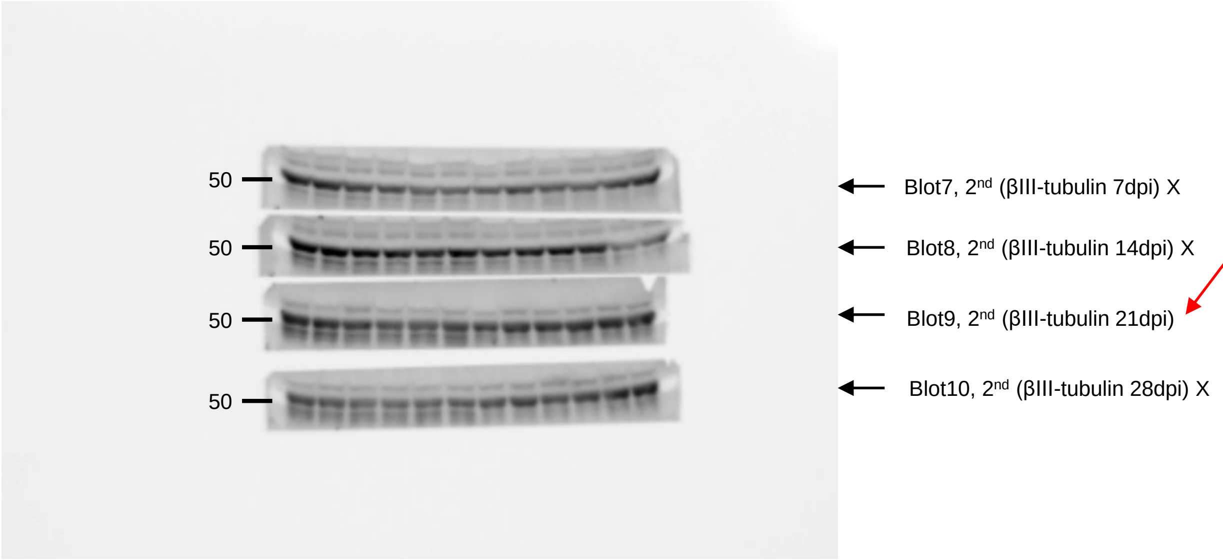

Original blot for the βIII-tubulin detection as internal marker for ATF4 detection in Fig. 3C.

Original blot images for Fig. 3C, continued.
For relative quantification and normalization of ATF4, blots were first probed with anti-ATF4 antibody (top) and after the detection of ATF4 by chemiluminescence, HRP conjugated to secondary antibody was inactivated with hydrogen peroxide treatment (15% H_2O_2 in PBS, for 30 min). After confirmation of the disappearance of chemiluminescence signal, blots were then secondarily probed with anti-βIII tubulin antibody for the detection of βIII tubulin as an internal marker.
