# Supplementary material for: Enhanced phosphorylation of PERK in primary cultured neurons as an autonomous neuronal response to prion infection
Source: PLoS One. 2020 Jun 1;15(6):e0234147. doi: 10.1371/journal.pone.0234147 (PMC7263615; doi:10.1371/journal.pone.0234147)
Supplement: Attachment — Submitted filename: Response to Reviewers_0503_MT.pdf [file pone.0234147.s007.pdf]

PONE-D-20-03488

Response to Reviewers

Thank you very much for reviewers and the editor for your careful reading of our manuscript. We tried to address concerns pointed out by reviewers as possible. We hope that our modifications are sufficient to wipe out your concerns.

Answers to comments from reviewer 1.

Thank you for your careful reading of our manuscript and helpful comments. Followings are our answers and changes that we have made in response to the reviewer's comments.

1. *The authors measured neuron density as NeuN positive nuclei per 0.01 mm$^2$. I wonder what the percentage of NeuN positive cells on the total DAPI positive cells is, since it seems that only few cells are positive to NeuN in fig S1.*

   Ans: Primary neuronal cultures contain large number of presumably dead cells which show shrunk and condensed nuclei. Such DAPI-positive condensed nuclei (DAPI-positive area ≤ 80 µm$^2$ in S1 Fig) were about 70% in CxN at 21 dpi and about 50% in ThN. We assume this is the main reason why NeuN-positive cells in the total DAPI-positive stains appeared to be low percentage in S2 Fig (the same as S1 Fig in the submitted manuscript).

   We analyzed again the presence of NeuN-positive neurons, GFAP-positive astrocytes, and NeuN- and GFAP- double negative cells with or without Ara-C treatment at 21 dpi, as suggested by reviewers 2 and 3. We removed presumably dead cells (Cells with DAPI-positive area ≤ 80 µm$^2$ and NeuN- and GFAP- double negative) from the cell count. NeuN-positive, GFAP-positive, and NeuN- and GFAP-double negative cells in Ara-C treated, mock-infected CxN were 89.3%, 3.5%, and 7.2%, respectively. Those in Ara-C treated, mock-infected ThN were 89.4%, 1.5%, and 9.1%, respectively. We have added these results as S1 Fig and following explanation to lines 266 to 270 in the revised version with track changes: "Proportion of neuronal cells in primary cultures were shown in S1 Fig. NeuN-positive, GFAP-positive, and NeuN- and GFAP-double negative cells were 89.3%, 3.5%, and 7.2%, respectively in AraC treated, mock-infected CxN. GFAP-positive cells were 13.8% in CxN without AraC treatment, indicating successful reduction of astrocyte growth in our CxN. Similar tendency was observed in ThN."

2. *Phosphorylation of PERK was enhanced in prion-infected cortical neurons as shown by immunoblot analysis. As normally phosphorylation level of PERK and eIF2alpha is*

*expressed as ratio between the phosphorylated and total protein signal, I suggest to express the data in figure 3 as ratio p-PERK/PERK and p-eIF2α/eIF2α.*

Ans: As suggested by the reviewer, ratios of p-PERK to total PERK and p-eIF2α to eIF2α were calculated and data were added as S3 Fig. Although differences were not statistically significant, p-PERK/total PERK ratios tended to be higher in prion-infected CxN and ThN than mock-infected CxN and ThN. In contrast to ratios of p-PERK/total PERK, those of p-eIF2α/ total eIF2α did not differ between prion-infected and mock-infected primary neurons. These are consistent with the finding of this paper that the phosphorylation of PERK was enhanced in prion-infected cortical neurons, there was no sign of activations of downstream unfolded protein response (UPR) in the PERK-eIF2α pathway. We have added explanation on the ratios of p-PERK to total PERK and p-eIF2α to eIF2α to lines 366 to 371 in the revised version with track changes as follows: "Ratios of ratios of p-PERK to total PERK and p-eIF2α to eIF2α were calculated (S3 Fig). Although differences were not statistically significant, p-PERK/total PERK ratios tended to be higher in prion-infected CxN and ThN than mock-infected CxN and ThN. In contrast to ratios of p-PERK/total PERK, those of p-eIF2α/ total eIF2α did not differ between prion-infected and mock-infected primary neurons.".

3. Typographical errors and embarrassed mistakes that reviewer 1 kindly pointed out have been corrected

- *line 28: alternations* >> alterations
- *line 109 autonomaous neurnal* >> autonomous neuronal
- I*n line 283 is SE or SD?*: "SE" is correct.
- *The percentage of* $PrP^{Sc}$ *positive ThN is 54.8% (line 274) or 52.8% (line 422)*: 52.8% in line 422 has been corrected to 54.8%
- line *503: *p<0.001* >> ***p<0.001

Answers to comments from reviewer 2.

Thank you for your careful reading of our manuscript and helpful comments. Followings are our answers and changes that we have made in response to the reviewer's comments.

1. *Why does prion infection activate only PERK phosphorylation, but not the downstream steps of the pathway? The authors do not offer much speculation or experimental evidence on this issue.*

   Ans: the reviewer questioned the reason why the downstream of PERK phosphorylation in UPR was not activated. As we described in the paragraph started at line 558 in the submitted version, there are three possibilities but those are a kind of general thought; $PrP^{Sc}$ level, duration of $PrP^{Sc}$ production, and involvement of glial cells. In the further study we will address these possibilities and we believe that the prion-infected primary neuronal cultures, in which PERK phosphorylation, the initial step of UPR pathway, is induced as autonomous neuronal responses to prion propagation, will be useful tool for further analysis of events involved in neurodegeneration in prion diseases.

   Recently, Smith et al reported that change in astrocyte secretome by UPR-response in astrocytes as the cause of non-neuron autonomous factor for neurodegeneration in prion diseases (Smith et al., Neuron, 2020), however, change in astrocytes by prion-induced UPR, which may cause the neurodegeneration, remains to be elucidated. Fang et al reported that $PrP^{Sc}$ caused acute toxicity in excitatory synapses through glutamate receptor and p38 MAPK activation, which is not related to UPR (Fang et al., PLoS Pathogen, 2018). We have added discussion related to these recent findings to lines 580 to 583 and 610 to 613, respectively, in the revised version with track changes.

2. *Why does PERK phosphorylation increase only in cortical and not thalamic neurons? This calls into question the generality of the results.*

   Ans: the reviewer asked if PERK phosphorylation is induced in prion-infected ThN. We did not show the results in the submitted version but we had analyzed induction of p-PERK in prion-infected ThN. Compared to the prion-infected CxN, ThN produced lower numbers of p-PERK granules (for instance, prion-infected CxN showed as much as nearly 30 p-PERK granular stains per prion-infected cells [Fig. 5B]; however, prion-infected ThN showed as much as less than ten p-PERK granular stains per prion-infected cells). However, $PrP^{Sc}$++ cells possessed higher p-PERK granular stains than mock-infected cells ($p < 0.05$). Thus, the induction of PERK phosphorylation occurs both in prion-infected CxN and ThN. We have added these

data as S5 Fig and following sentence to lines 456 to 457 in the revised version with track changes: “The acceleration of PERK phosphorylation was also observed in $PrP^{Sc}$++ neurons of ThN (S5 Fig)”

3. *The authors do not do a very good job reconciling their results with those of Mallucci or Fang et al. (2018).*

Ans: We appreciated the reviewer’s comment. Recently, Smith et al reported that change in astrocyte secretome by UPR-response in astrocytes as the cause of non-neuron autonomous neurodegeneration in prion diseases. Fang et al reported that $PrP^{Sc}$ caused acute toxicity in excitatory synapses through glutamate receptor and p38 MAPK activation, which is not related to UPR (Fang et al., PLoS Pathogen, 2018). We have added discussion related to these recent findings to lines 580 to 583 and 610 to 613, respectively, in the revised version with track changes.

Lines 580 to 583: “Recently, Smith et al. reported that changes in astrocyte secretome by UPR-response in astrocytes as the cause of non-neuron autonomous neurodegeneration in prion diseases, although changes in astrocytes by prion-induced UPR, which may directly cause the neurodegeneration, are not fully understood [62]”

Line 610 to 613: “Alternatively, it is reported that $PrP^{Sc}$ causes acute toxicity in excitatory synapses through glutamate receptor and p38 MAPK activation, which is not presumably related to UPR [68]. Thus, analyses of earlier time points are also required to evaluate neuron-autonomous responses to prion infection.”

4. *What is the proportion of neurons in culture? When was AraC added to the cultures? These primary cultures probably contain a mixture of cell types, and non-neuronal cells may account for up to 30% of total cells (Ref. #17). Therefore, immunoblot analysis for protein levels (Fig. 3) will represent the responses of multiple cell types, and not just neurons. Even though the authors normalized their Western blot data using βIII tubulin, this will only equalize the proportion of neuronally-derived protein in each sample, but not remove the effect of non-neuronal cells. This problem is compounded in the normalization for the pre- and post-synaptic markers (Fig. 4), since the proportion of neuronal vs. non-neuronal cells is likely changing over time even in the absence of prion infection.*

Ans: the reviewer asked the proportion of neuronal cells in our primary neuronal culture. We have analyzed again the presence of NeuN-positive neurons, GFAP-positive astrocytes, and NeuN- and GFAP- double negative cells with or without Ara-C

treatment at 21 dpi. NeuN-positive, GFAP-positive, and NeuN- and GFAP-double negative cells were 89.3%, 3.5%, and 7.2%, respectively in Ara-C treated, mock-infected CxN. Proportion of GFAP-positive cells were 13.8% without Ara-C treatment, indicating successful reduction of astrocyte growth in our primary CxN. NeuN-positive, GFAP-positive, and NeuN- and GFAP-double negative cells were 81.4%, 6.2%, and 12.4%, respectively in Ara-C treated, prion-infected CxN, also indicate the reduction of astrocyte growth by Ara-C treatment. Reduction of astrocyte growth was also observed in primary ThN cutlures treated with Ara-C. We have added these results as S1 Fig and following explanation to lines 266 to 270 in the revised version with track changes: "Proportion of neuronal cells in primary cultures were shown in S1 Fig. NeuN-positive, GFAP-positive, and NeuN- and GFAP-double negative cells were 89.3%, 3.5%, and 7.2%, respectively in AraC treated, mock-infected CxN. GFAP-positive cells were 13.8% in CxN without AraC treatment, indicating successful reduction of astrocyte growth in our CxN. Similar tendency was observed in ThN."

5. *The authors used only the phospho-PERK (Thr980) (16F8) antibody, but this product is recommend only for Western blots. This raises a question about the specificity of the IF staining using this antibody.*

   Ans: the reviewer asked the specificity of anti-phospho-PERK (Thr980) (16F8) that was used in this study in IFA. We had examined if this antibody works in IFA using Neuro2a cells treated with tunicamycin that is known to induce UPR. Fluorescent granular signals stained by this antibody appeared more in tunicamycin-treated Neuro2a cells (5.0 µg/ml) than in untreated Neuro2a cells, indicating that the specificity of reaction of this antibody in IFA. We have added the results as S4 Fig and related explanation to lines 441 to 444 in the revised version with track changes: "Specificity of anti-phospho-PERK antibody in IFA was confirmed using tunicamycin-treated Neuro2a cells; fluorescent granular signals stained by this antibody appeared more in tunicamycin-treated Neuro2a cells (5.0 µg/ml) than in untreated Neuro2a cells (S4 Fig)."

Answers to comments from reviewer 3.

Thank you for your careful reading of our manuscript and helpful comments. Followings are our answers and changes that we have made in response to the reviewer's comments.

1. *Evidence for this conclusion is very compelling, although the authors did not discuss in the manuscript how pure their neuronal cultures at the end really are. They provide references to earlier publications, but it is necessary to provide this data or at least discuss them.*
   Ans: The reviewer pointed out the proportion of neuronal cells during the experiments, which is the same as the comment by reviewer 2. We have analyzed again the presence of NeuN-positive neurons, GFAP-positive astrocytes, and NeuN- and GFPA- double negative cells with or without Ara-C treatment at 21 dpi. NeuN-positive, GFAP-positive, and NeuN- and GFAP-double negative cells were 89.3%, 3.5%, and 7.2%, respectively in Ara-C treated, mock-infected CxN. Proportion of GFAP-positive cells were 13.8% without Ara-C treatment, indicating successful reduction of astrocyte growth in our primary CxN. NeuN-positive, GFAP-positive, and NeuN- and GFAP-double negative cells were 81.4%, 6.2%, and 12.4%, respectively in Ara-C treated, prion-infected CxN, also indicate the reduction of astrocyte growth by Ara-C treatment. Reduction of astrocyte growth was also observed in primary ThN cutlures treated with Ara-C. We have added these results as S1 Fig and following explanation to lines 266 to 270 in the revised version with track changes: "Proportion of neuronal cells in primary cultures were shown in S1 Fig. NeuN-positive, GFAP-positive, and NeuN- and GFAP-double negative cells were 89.3%, 3.5%, and 7.2%, respectively in AraC treated, mock-infected CxN. GFAP-positive cells were 13.8% in CxN without AraC treatment, indicating successful reduction of astrocyte growth in our CxN. Similar tendency was observed in ThN."

2. *Page 8/Immunoblotting: Duration of PK digestion should be indicated.*
   Ans: According to the reviewer's comment, we have added "for 10 min at 37°C" to line 160 in the revised version with track changes, as the condition of PK digestion.

3. *Page 8/IF and page 18: mAb 8D5 should also be described in Materials and Methods, with clearly mentioning that no GndSCN step is done.*
   Ans: As suggested by the reviewer, we have added "while GdnSCN treatment was omitted for $PrP^{Sc}$-specific staining with mAb 8D5" to lines 169 to 170 in the revised version (Materials and Methods). We have also added "such as GdnSCN" to line 437 in the revised version with track changes.

4. *Fig. 1A/B: It would help to indicate in the figure and/or charts that A shows CxN and B ThN neurons.*

   Ans: As suggested by the reviewer, we have added "CxN" and "ThN" to the Fig. 1A and 1B.

5. *Fig 1: The rationale of relating quantification of $PrP^{Sc}$ to day 7 p.i. should be provided.*

   Ans: In this experiment, we would like to show rate of prion propagation in primary neurons after the establishment of prion infection. At day 0, $PrP^{Sc}$ in the inoculum, was detectable at 0 dpi. However, small portion of the inoculated prions initiated productive infection in the primary neuron. Thus, relative $PrP^{Sc}$ levels to day 0 does not show the true increase of $PrP^{Sc}$ that reflects prion propagation in neurons. Hence, we performed relative quantification of PrP-res to 7 dpi, when newly generated prions could be detected as PrP-res. We have added the reasons in the legend for Fig. 1 (Lines 290 to 291 in the revised version with track changes) as follows: "in order to show rate of prion propagation (detected as PrP-res) after the establishment of prion infection in the primary neurons."
